# Targeting and limiting surgery for patients with node-positive breast cancer

**DOI:** 10.1186/s12916-015-0385-5

**Published:** 2015-06-25

**Authors:** Abigail S. Caudle, Henry M. Kuerer

**Affiliations:** Department of Surgical Oncology, The University of Texas MD Anderson Cancer Center, 1515 Holcombe Blvd, Unit1484, Houston, TX 77230-1402 USA

**Keywords:** Axillary lymphadenectomy, Breast cancer, Neoadjuvant chemotherapy, Nodal metastasis, Sentinel lymph node, Targeted axillary dissection

## Abstract

The presence of axillary nodal metastases has a significant impact on locoregional and systemic treatment decisions. Historically, all node-positive patients underwent complete axillary lymph node dissection; however, this paradigm has changed over the last 10 years. The use of sentinel lymph node dissection has expanded from its initial role as a surgical staging procedure in clinically node-negative patients. Clinically node-negative patients with small volume disease found on sentinel lymph node dissection now commonly avoid more extensive axillary surgery. There is interest in expanding this role to node-positive patients who receive neoadjuvant chemotherapy as a way to restage the axilla in hopes of sparing women who convert to node-negative status from the morbidity of complete nodal clearance. While sentinel lymph node dissection alone may not accomplish this goal, there are novel techniques, such as targeted axillary dissection, that may now allow for reliable nodal staging after chemotherapy.

## Background

The presence of axillary lymph node metastases is the most significant prognostic predictor in breast cancer, and is often used to guide locoregional as well as systemic therapy decisions [1–3]. The surgical management of the axilla has undergone many changes with the development of effective systemic therapy and improvement in diagnostic tools. Many recent trials have resulted in national conversation about optimal nodal management with respect to diagnosis and therapy [4–10]. In clinically node-negative patients undergoing surgery as the first component of their breast cancer treatment, sentinel lymph node dissection (SLND) is the standard surgical approach to axillary staging. Multiple studies have demonstrated that a sentinel lymph node (SLN) can be identified in 93–99 % of patients with a false negative rate (FNR; i.e., number of patients in whom no cancer is seen in the SLN but metastases are identified in other axillary nodes divided by the total number of node positive patients) of 5–11 % [11, 12]. If the SLN is negative for metastases, then no further axillary surgery is required and the remaining lymph nodes can be left in place. While historically patients with a positive SLN underwent axillary lymph node dissection (ALND), this paradigm has changed in the last 10 years.

### Clinically node-negative patients with limited pathologic node-positive disease

**Fig. 1:**
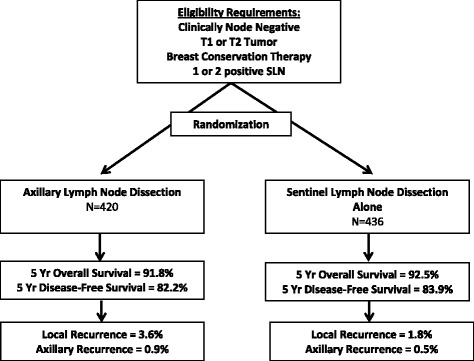
Schema for the ACOSOG Z0011 Trial [4, 14]. The ACOSOG Z0011 trial was designed to determine whether there was a difference in overall survival or locoregional recurrence in early breast cancer with one or two positive sentinel lymph nodes who underwent axillary lymph node dissection versus those that had no further axillary therapy

Several phase III, multicenter trials showing that ALND can be omitted in selected SLN-positive women have recently been reported with resulting changes in clinical practice [4–6, 13]. These are summarized in Table 1. The American College of Surgeons Oncology Group (ACOSOG) Z0011 trial was a multi-institutional, prospective non-inferiority trial [4, 14] which enrolled clinically node-negative patients with T1 or T2 tumors treated with breast conservation therapy (BCT) and adjuvant radiotherapy and were found to have one or two positive SLNs. Patients were randomized to completion ALND versus SLN alone and followed for evidence of disease recurrence and for overall survival. There were no differences in 5-year overall survival (91.9 % in ALND vs. 92.5 % in SLND alone, *P* = 0.24) or disease-free survival (82.2 % vs. 83.8 %, *P* = 0.13). In patients randomized to ALND, additional positive non-SLNs were identified in the axillary specimen in 27 % of cases. The investigators concluded that ALND could be safely omitted in clinically node-negative patients with T1 or T2 tumors undergoing BCT with one or two positive lymph nodes. The majority of patients in this trial had post-operative adjuvant whole-breast radiotherapy and systemic therapy without specific directed nodal radiotherapy to the axilla [15] (Fig. 1).

**Table 1:** Clinical trials evaluating axillary lymph node dissection in clinically node-negative patients. Summary of trials evaluating the role of axillary lymph node dissection in patients presenting with no clinical evidence of axillary lymphadenopathy

Trial	Breast surgery	Number of positive sentinel lymph nodes allowed	Randomization groups	Number	5-year locoregional recurrence	5-years overall survival
ACOSOG Z0011 [4, 14]	BCT	1-2	ALND	420	1.6 %	91.9 %
			No ALND ^a^	436	3.1 %	92.5 %
AMAROS [5]	BCT or Mastectomy	No limit	ALND	744	Axillary recurrence 0.43 %	93.3 %
			Axillary RT	682	1.19 %	92.5 %
IBCSG 23-01 [6]	BCT or Mastectomy	No limit- all metastases had to be ≤2 mm	ALND	464	2.4 %	97.6 %
			No ALND ^a^	467	2.8 %	97.5 %

^a^No axillary RT allowed

BCT, Breast conservation therapy; ALND, Axillary lymph node dissection; RT, Radiotherapy

A similar European trial, the EORTC 10981-22023 AMAROS (After Mapping of the Axilla, Radiotherapy or Surgery?) trial, was also a multi-institutional trial enrolling clinically node-negative patients with positive SLNs [5]. The trial started with eligibility criteria of a unifocal tumor <3 cm, which was later expanded to include tumors up to 5 cm and those with multifocal disease. Similar to ACOSOG Z0011, a positive SLN was considered positive by standard hematoxylin and eosin staining with isolated tumor cells considered negative. They randomized 744 patients to ALND and 681 patients to axillary radiotherapy. Unlike the Z0011 trial, the type of breast surgery was not dictated by the protocol, so patients undergoing mastectomy were eligible for enrollment (17 % of the ALND cohort and 18 % of the axillary radiotherapy group). In the ALND group, 33 % (220/672) had additional positive non-SLNs identified. They reported a 5-year axillary recurrence rate of 0.43 % after ALND and 1.19 % in the axillary radiotherapy group. The study was underpowered to show statistical assurance of non-inferiority due to the low number of events. Because patients were enrolled and randomized before surgery, the trial included results on 3131 SLN-negative patients with a 0.72 % 5-year axillary recurrence rate. There were no differences in 5-year disease-free survival (86.9 % in ALND vs. 82.7 % in radiotherapy, *P* = 0.18) or 5-year overall survival (93.3 % in ALND vs. 92.5 % in radiotherapy, *P* = 0.34). They also reported that clinical evidence of lymphedema was higher in the ALND group at 5 years (23 % compared to 11 %, *P* <0.0001) as well as the proportion of patients with a ≥10 % increase in arm circumference (13 % vs. 6 %, *P* = 0.0009). Interestingly, despite these changes, there were no differences in quality of life scores.

The last trial that has led to a change in practice from ALND to limited surgery with SLND is the International Breast Cancer Study Group (IBCSG) 23-01 trial [6]. This phase 3 non-inferiority trial randomized clinically T1/2, N0 patients with micrometastases identified in SLNs to ALND versus no further surgery. The trial did not mandate any specific breast procedure so it included patients who underwent mastectomy and BCT and could have any number of positive SLNs as long as all metastases were ≤2 mm [14]. This trial varies from the previous ones in that a significant proportion of patients received no radiotherapy or partial breast radiotherapy that would not have incorporated the nodal region. Only 9 % of patients in each group underwent mastectomy and none received adjuvant radiation. In the remaining 91 % of patients who underwent BCT, 19 % of both groups received intra-operative radiotherapy alone, 70 % received standard adjuvant whole breast radiation therapy, and 9 % of the ALND group and 8 % of the no ALND group received a combination of intra-operative and whole breast radiation therapy, while 2–3 % of the groups did not receive any radiotherapy. Possibly reflecting the fact that only patients with micrometastases were eligible for enrollment, only 13 % of patients in the ALND group had additional positive non-SLNs. Locoregional recurrences were similar in the two groups – 2.4 % (11/464) of the ALND group versus 2.8 % (13/467) of patients without ALND. Further, 5-year disease-free survival was 84.4 % in the ALND cohort compared to 87.8 % in the group without ALND (*P* = 0.16).

While each of these three trials had different study designs and eligibility criteria, they all reflect the same notion that patients with clinically occult nodal metastases found by SLND can safely avoid completion ALND with equivalent oncologic outcomes.

### Timing of SLND in patients undergoing neoadjuvant chemotherapy

Neoadjuvant chemotherapy (NCT) is increasingly used in node-negative breast cancer patients with the goal of downsizing the tumor, which may facilitate BCT. The preoperative administration of chemotherapy allows for assessment of *in situ* tumor response, thus identifying agents with no efficacy early so that the patients can be spared unnecessary toxicity. In addition, complete pathologic response (pCR) is now recognized as a surrogate for long term outcomes, which has made the neoadjuvant approach a valuable research platform [16, 17]. Another benefit of NCT is that 40–75 % of patients presenting with clinically occult or biopsy proved-positive lymph nodes will convert to pathologic lymph node-negative when the nodes are removed at surgery [18–20]. Thus, SLND can lead to different results (and resulting adjuvant therapies) depending on whether it is performed before or after NCT. Advocates for performing upfront SLND before initiating chemotherapy contend that SLN identification is more successful before chemotherapy and that complete nodal staging is important to treatment planning [21]. However, this approach commits all women, even if the SLN is negative, to two surgical procedures. Furthermore, it commits women with clinically occult nodal disease to ALND even though the nodal metastases would have been easily eradicated with chemotherapy. In addition, performing SLND after NCT reveals the nodal status after NCT, which is a better prognostic indicator than the identification of occult nodal metastases pre-NCT [22]. In one study from the MD Anderson Cancer Center, the SLN identification rate was not altered by the order of therapy (98.7 % if surgery first vs. 97.4 % if SLN performed after NCT) with similar FNRs (4.1 % in surgery first cohort vs. 5.8 % in NCT) [23]. After stratification for tumor size, the probability of discovering positive SLNs was lower if performed after NCT as opposed to before chemotherapy, which resulted in fewer patients requiring ALND.

### Patients presenting with clinically node-positive disease

**Fig. 2:**
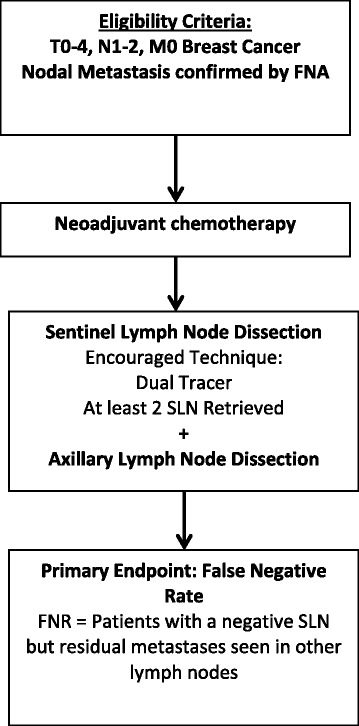
ACOSOG Z1071 Trial [7]. The ACOSOG Z1071 trial was designed to test the reliability of sentinel lymph node dissection to restage the axillary lymph nodes after neoadjuvant chemotherapy in patients presenting with clinically positive lymph nodes

The role of SLND in patients who present with clinically involved and biopsy proven lymph nodes and have a clinical response to NCT is currently under review. Since 40–75 % of patients have eradication of their nodal disease [18, 20, 24, 25], there is considerable interest in finding reliable methods to restage the axilla in hope of sparing a significant percentage of patients from the morbidity of ALND. There are concerns, however, that SLND may not be accurate in this setting – single institution reports have shown unacceptably high FNRs of 15–30 % [26–29]. The ACOSOG Z1071 trial was designed to test the hypothesis that SLND performed with a standardized surgical approach would accurately assess nodal response after chemotherapy. The study enrolled women with clinical T0-4 N1-2 M0 breast cancer with nodal metastases confirmed by needle biopsy. After completing neoadjuvant chemotherapy, enrolled patients underwent SLND followed by completion ALND in order to assess the FNR (Fig. 2). The study was designed with a prespecified 10 % success threshold for FNR in these patients. The overall nodal conversion rate was 41.1 %, but the trial confirmed previous reports that tumor biology reflected in receptor subtype influenced the probability of nodal conversion [24]. While only 21.1 % (67/317) of patients with hormone-positive disease achieved a nodal pCR, 49.4 % (84/170) of patients with triple negative disease and 64.7 % (134 /207) of those with HER2 amplified disease had nodal conversion.

SLNs were detected in 92.9 % of patients. In multivariate analysis, only the use of a single tracer instead of dual tracers increased the likelihood of not finding a SLN (OR, 3.82; 95% CI, 1.47–9.92). Clinical T or N stage, patient age, body mass index, or duration of chemotherapy did not influence the ability to find at least one SLN [30]. In 525 cN1 patients who had at least two SLNs excised, the reported FNR was 12.6 % (90 % CI, 9.85–16.05 %). One finding from the trial was that surgical technique was crucial to SLND accuracy in this setting. In contrast to the available literature from clinically node-negative patients that suggests that use of a single tracer is acceptable [31, 32], the Z1071 trial had very different results. The FNR rate improved dramatically from 20.3 % (95 % CI, 11–32.8 %) when a single tracer was used to 10.8 % (95 % CI, 7.2–15.3 %) with the use of dual tracers (*P* = 0.05). They also showed that the FNR improved with the number of SLNs removed from 31 % (17/54) when only one node was removed, to 21 % (19/90) when two nodes were removed, and to 9.1 % (20/220) when three or more nodes were removed [7].

Since publishing of the trial primary endpoints, the authors have further scrutinized the data for insights into patient populations or technical aspects which could improve the accuracy of SLND in predicting nodal conversion. Central review of post-chemotherapy ultrasounds was performed in 611 patients to determine if ultrasonography could predict nodal response. An abnormal ultrasound after NCT was reasonably reliable – 71.8 % of those patients did indeed have positive nodes on surgical pathology. However, an ultrasound that showed normal-appearing nodes was less accurate, as 56.5 % of those patients actually had residual nodal disease. The authors suggest that the use of ultrasound in this setting might not accurately stage the axilla alone, but might serve as a complement to SLND. If SLND had only been performed on trial participants who had a normal appearing ultrasound after chemotherapy, the FNR would be 9.8 % [8].

**Fig. 3:**
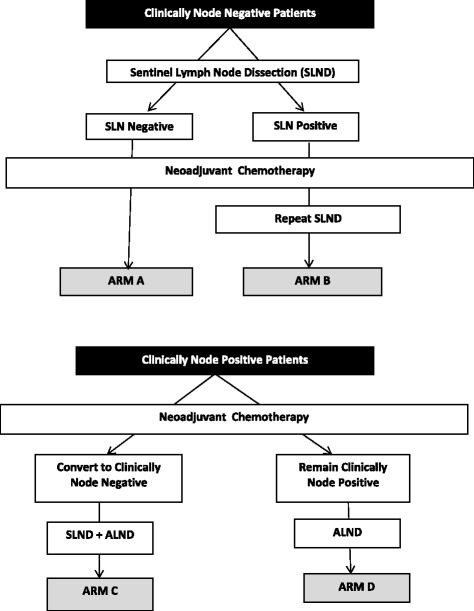
SENTINA Trial [9]. The SENTINA trial was designed to evaluate the relationship of sentinel lymph node dissection in patients who received neoadjuvant chemotherapy; the study arms are depicted below

The more comprehensive SENTINA (SENTinel NeoAdjuvant) study was designed to evaluate the optimal timing of SLND in patients receiving NCT [9]. There were four arms in the trial: (A) clinically node-negative patients who underwent SLND before NCT, a portion of whom were then moved to arm (B) if they had a positive SLN, and then had a second SLND after NCT. The third arm (C) consisted of clinically node-positive patients who converted to clinically negative after NCT and then underwent SLND to restage the axilla followed by ALND. The remaining arm (D) consisted of clinically node-positive patients who remained clinically positive after NCT and underwent ALND (Fig. 3). In contrast to the ACOSOG Z1071 trial, patients in the SENTINA study did not have nodal metastases confirmed by needle biopsy. The authors showed that SLNs could be detected in 99.1 % before NCT (Arm A); however, among patients who had nodal metastases identified by a SLND prior to NCT, a second SLND procedure (Arm B) was only successful in 60.8 % demonstrating that patients should only undergo one SLN procedure for staging. Arm C focused on the possibility of accurately restaging the axillary nodes after NCT in clinically node-positive patients. The authors report an overall FNR for SLND in these patients of 14.2 %, with findings similar to the Z1071 trial that the FNR was lower when more SLNs were retrieved and dual tracers were used. Of note, one important aspect of the trial is that they did not require pathologic confirmation of lymph node involvement. The FNR for SLND in the 149 patients who had biopsy-confirmed metastases was 19 % compared to 12.3 % in the 443 patients who were classified as node-positive without pathologic confirmation.

Finally, the sentinel node biopsy following neoadjuvant chemotherapy Canadian multi-institutional study (SN FNAC) of patients with needle biopsy-proven node-positive breast cancer [10], including 153 patients, reported that the accuracy of SLND could be improved with the use of immunohistochemistry (IHC). In their trial, if macrometastases (>2 mm) were considered positive and micrometastases or isolated tumor cells were considered negative, the FNR would be 13.3 % (11/83) and was decreased to 8.4 % (7/83) if micrometastases and isolated tumor cells were considered positive. This study also showed that the use of dual tracers and the retrieval of two or more SLNs were crucial to improving accuracy. The investigators did an unplanned interim analysis after the ACOSOG Z1071 results were released. Given the similarity in their results and because of slow accrual, they made the decision to close the trial before meeting their target accrual. The ACOSOG Z1071 authors have also looked at the contribution of IHC to improve SLND accuracy. While the trial defined metastases as those seen on standard hematoxylin and eosin staining, IHC was available on 470 patients. Using the IHC results, an additional 17 patients were found to have positive SLNs, which improved the FNR to 8.7 % (95 % CI, 5.6–11.8) [33]. The results of these trials are summarized in Table 2.

**Table 2:** Clinical trials evaluating the accuracy of sentinel lymph node dissection in clinically node-positive patients. Summary of trials designed to evaluate the accuracy of sentinel lymph node dissection to assess axillary nodal response to therapy in clinically node-positive patients who receive neoadjuvant chemotherapy

Trial	ACOSOG Z1071 [7, 33]	SENTINA (Arm C) [9]	SN FNAC [10]
Nodal eligibility criteria	cN1-2	cN1-2	cN1-2
	Endpoints reported for cN1 ^a^		
Biopsy required to confirm metastases?	Yes	No	Yes
Number of patients	cN1 = 603	592	153
	cN2 = 34		
Overall FNR (No IHC)	12.6 % ^a^	14.2 %	13.4 %
FNR with IHC	8.7 % ^a^	Not reported	8.4 %
FNR depending on mapping agents			
One agent	20.3 %	16 %	16 %
Dual agents	10.8 %	8.6 %	5.2 %
FNR by number of SLNs			
One SLN	31 %	24.3 %	18.2 %
Two SLNs	21.1 %	18.5 %	≥2 SLNs = 4.9 %
Three or more SLNs	9.1 %	4.9 %	

^a^Limited to patients classified as cN1 with ≥2 sentinel lymph nodes removed

FNR, False negative rate; IHC, Immunohistochemistry; SLN, Sentinel lymph node

While these trials did not meet their success thresholds, there has been considerable national debate about how to move forward in these efforts to accurately identify women who might safely avoid ALND after chemotherapy. One aspect being explored is actually based on a very logical principle – perhaps the best lymph node to evaluate after chemotherapy in order to determine response is the node that had confirmed metastases before therapy, i.e., the biopsied node. In the ACOSOG Z1071 trial, 170 patients had a clip placed in the biopsied node similar to the way breast primary tumor sites are marked after biopsy. In 107 patients in whom the SLN retrieved was also documented to be the biopsy-proved clipped node the FNR improved to 6.8 % (95 % CI, 1.9–16.5). The clipped node was identified as a part of the remaining axillary contents in 34 patients and was not identified in 29 patients [33].

### Development of novel procedures to increase accuracy and target nodal disease after NCT

The intuitive concept that the best node to evaluate response after NCT is the individual node that had been proved to contain metastases by needle biopsy at diagnosis before NCT has been explored at MD Anderson Cancer Center and at the Netherlands Cancer Institute [34, 35]. In 2011, MD Anderson investigators established a prospective registry study enrolling patients with biopsy-confirmed nodal metastases with a clip placed in the biopsied lymph node. The trial was designed to test the hypotheses that the pathologic changes in the clipped node with metastases accurately reflect the response to therapy in other nodes. This data was recently presented at the Society of Surgical Oncologists Annual meeting [34], and publication of this data is anticipated soon. Preliminary data shows that specific evaluation of the clipped node with documented metastases before NCT in addition to SLND lowers the FNR over SLND alone. In 25 % of cases evaluated, the clipped node could not be identified as a sentinel node using dual mapping agents or palpation. That is, if the SLND procedure was performed alone, the node that had been proven to have metastases prior to NCT would have been left in the patient and not tested in a quarter of cases. While this data is encouraging with respect to increasing the accuracy of identifying residual disease, the problem remained whether a clipped node could be selectively localized and removed intra-operatively. This challenge was answered with the development of targeted axillary dissection (TAD) [36], which involves removal of the node with known metastases (containing the clip) in addition to removal of the nodes most likely to harbor disease (the SLNs). Similar to the techniques for breast tumor localization [37, 38], an I^125^ seed is placed in the clipped node under ultrasound guidance 1–5 days before surgery, followed by radioisotope injection either preoperatively or intra-operatively. At the time of surgery, the surgeon uses a gamma probe to identify the node containing the clip and the seed, and removes it. The surgeon proceeds to remove any other blue nodes and uses the gamma probe on the technetium settings to identify any remaining sentinel nodes. The seed has been successfully retrieved in all cases to date and does not interfere with SLND. Given the low FNR when this approach is used, it may be reasonable to consider TAD for staging of the axilla after NCT in selected patients with plans to omit ALND if no residual disease is identified.

The initial results of the Netherland Cancer Institute Marking Axillary Lymph Nodes with Radioactive Iodine procedure in 100 patients with needle biopsy-proven metastases for axillary staging after NCT was also recently published [35]. A radioactive seed is placed as the time of the initial biopsy if metastases are confirmed and left in place through NCT. At surgery, the surgeon uses a gamma probe to excise the node to assess response. In this study, the radioactive node was identified in 97 % and all patients underwent completion dissection to determine the FNR, which was found to be 7 %. This was a stand-alone procedure with completion ALND and no lymphatic mapping or SLND was attempted in this study. The authors also concluded that the procedure might be useful in tailoring axillary therapy among patients who present with nodal metastases. Leaving the radioactive seed in place for 3–6 months during NCT would not likely to be feasible in the USA. Marking of the nodes with documented metastases using India ink at diagnosis has also been proposed [39], but many surgeons are concerned that this might require more dissection of healthy lymphatics to identify and retrieve these nodes after NCT compared with more targeted methods. Efforts are now underway to identify alternative approaches to localize nodes with proven metastases using novel localizing methods.

### Ongoing and upcoming clinical trials addressing axillary disease management

#### NSABP-51/RTOG 1304 trial

With the acknowledgement that selected clinically node-positive patients who have a response to NCT may not undergo ALND in the future, cooperative groups are organizing trials to evaluate the optimal locoregional treatment for patients. One such trial, NSABP-51/RTOG 1304, is currently enrolling patients with biopsy-proven node-positive (N1) disease who undergo NCT and have no residual nodal disease (by SLND or ALND), and randomizes them to axillary radiation versus no axillary radiation. The primary endpoints will be recurrence and survival, but information on toxicity, effect of radiation on cosmetic outcome, and quality of life will also be collected [40].

#### ALLIANCE trial A11202

Another cooperative group trial is enrolling patients with biopsy-proven N1 disease who do not achieve a nodal pCR with NCT [41]. The goal of the Alliance A11202 trial is to compare the efficacy of ALND plus radiation to radiation alone clinically node-positive patients who remain node-positive at SLND after NCT. The primary end points of the trial are locoregional recurrence and survival; however, there is a strong correlative component of the trial dedicated to lymphedema that should help delineate the differences in toxicity between axillary surgery and radiation together versus radiation alone.

#### MD Anderson Trial 2013-0877

If it is feasible to identify abnormal axillary nodes and prove metastases by percutaneous needle-biopsy before NCT, might it be possible to do this after NCT and potentially spare patients from any axillary nodal surgery? This concept and hypothesis is being tested in MD Anderson trial 2013-0877, which is designed as a study to correlate fine needle aspiration to surgical excision to assess for eradication of nodal metastases after NCT in breast cancer.

## Conclusions

The evaluation and management of axillary lymph nodes is critical in breast cancer with impact on locoregional as well as survival outcomes. ALND can be extremely morbid for patients and adversely impact quality of life. While ALND has historically been the standard approach to patients with nodal metastases, emerging data has identified patients at low risk for regional recurrence who may be spared the morbidity of this procedure in the setting of appropriate multidisciplinary care. The omission of ALND in clinically node-negative patients with nodal metastases discovered by SLND has been incorporated broadly into clinical practice, although it is unclear if the inclusion of axillary radiotherapy adds substantial benefit. The ability of SLND to accurately identify patients with a nodal pCR after NCT is still being evaluated with the recognition that technical aspects are crucial to the reliability of the test. Targeted axillary dissection, or SLND in addition to specific removal of the sampled node (containing a clip), may be a way forward in accurately restaging the axilla, thus identifying women who do not benefit from completion ALND even among patients who present with biopsy-proven nodal metastases. Moving forward, the safety and efficacy of selective omission of ALND among patients who convert from biopsy-proven node-positive breast cancer to pathologic negative disease after NCT must be systematically studied. It is anticipated that systemic agents for breast cancer will further advance and the future holds the potential for even elimination of axillary surgery in patients with node-positive breast cancer after NCT by incorporating improved imaging modalities with or without percutaneous sampling of tissues for eradication of disease.

## Abbreviations

ACOSOG: American College of Surgeons Oncology Group
ALND: Axillary lymph node dissection
BCT: Breast conservation therapy
FNR: False negative rate
IHC: Immunohistochemistry
NCT: Neoadjuvant chemotherapy
pCR: Complete pathologic response
SLN: Sentinel lymph node
SLND: Sentinel lymph node dissection
